# S-Glutathionylated Serine Proteinase Inhibitors as Biomarkers for Radiation Exposure in Prostate Cancer Patients

**DOI:** 10.1038/s41598-019-50288-9

**Published:** 2019-09-24

**Authors:** Leilei Zhang, Jie Zhang, Zhiwei Ye, Yefim Manevich, Danyelle M. Townsend, David T. Marshall, Kenneth D. Tew

**Affiliations:** 1 Departments of Cell and Molecular Pharmacology and Experimental Therapeutics, Medical University of South Carolina, Charleston, South Carolina USA; 2 Departments of Pharmaceutical and Biomedical Sciences, Medical University of South Carolina, Charleston, South Carolina USA; 3 Departments of Radiation Oncology, Medical University of South Carolina, Charleston, South Carolina USA

**Keywords:** Tumour biomarkers, Post-translational modifications

## Abstract

In biological tissues, radiation causes the formation of reactive oxygen species (ROS), some of which lead to sequential oxidation of certain protein cysteine residues. Resultant cysteinyl radicals are subject to post-translational modification through S-glutathionylation. The present clinical trial was designed to determine if S-glutathionylated serine protease inhibitors (serpins) in blood could be used as biomarkers of exposure to radiation. 56 male prostate cancer patients treated with radiotherapy were enrolled in the trial and levels of S-glutathionylated serpins A1 and A3 were assessed by immunoblotting. Patients were classified into three groups: (1) external beam radiation therapy (EBRT); (2) brachytherapy (BT); (3) both EBRT and BT. Prior to treatment, baseline plasma levels of both unmodified and S-glutathionylated serpins were similar in each group. We identified elevated plasma levels of S-glutathionylated serpin A1 monomer, trimer and serpin A3 monomer in patient blood following radiation. Maximal increased levels of these S-glutathionylated serpins were correlated with increased duration of radiotherapy treatments. We conclude that it is practical to quantify patient plasma S-glutathionylated serpins and that these post-translationally modified proteins are candidate biomarkers for measuring radiation exposure. This provides a platform for use of such biomarkers in trials with the range of drugs that, like radiation, produce ROS.

## Introduction

According to the Personalized Medicine Coalition, 25 of 59 (42%) of all new FDA approved drugs in 2018 were tailored towards individual patients, primarily as a consequence of using biomarkers as predictors of response^1^. The present trend in development of targeted anticancer drugs means that early clinical testing can be facilitated by the inclusion of reliable biomarkers that can be used to predict treatment efficacy, or treatment associated toxicities. Under biological conditions, the majority of small molecule cancer drugs^2^, as well as radiation^3^, produce chemical electrophiles that generate a variety of reactive oxygen species (ROS) and free radicals. In turn, these impact redox homeostasis in tissue and blood compartments. When exposed to ROS, cysteine residues in certain proteins can become oxidized with the initial formation of a cysteinyl radical, sequentially leading to a disulfide with the cysteine of glutathione (GSH), a process referred to as S-glutathionylation^4^. This reversible post-translational modification has the initial effect of protecting the cysteine from sequential oxidation to a sufenate, sulfinate or sulfonate, the latter two states invariably leading to its proteosomal degradation^5^. S-glutathionylation is a cyclical event where the forward reaction may be catalyzed by glutathione S-transferase P (GSTP^6^) and the reverse by glutaredoxin^7^. At such time as ROS levels recede, deglutathionylation can occur. Under such reversible conditions the half-life of the post-translational modification approximates 4 hours^8^. In many instances, in addition to the protective effect, S-glutathionylation can alter the structure and/or function of the protein and modify a variety of biologically significant protein:protein interactions^9^. In the case of serine protease inhibitors (serpins), oxidation of sensitive cysteine residues can result in inhibition of their activities. However, where endogenous or exogenous ROS are present, S-glutathionylation of either Cys256 of serpinA1 or Cys263 of serpin A3^10,11^ can prevent their oxidation and protect enzyme activities^12,13^. Serpins are found in both the bloodstream and in tissues and can serve a broad range of physiological functions. Redox regulation of members of the serpin family can play a role in regulation of myeloproliferation and hematopoietic progenitor cell mobilization^14^.

We have previously considered whether serpin S-glutathionylation could be used as a surrogate biomarker. Our earlier work in mice exposed to various cancer drugs^15^, as well as measurements in buccal cell samples taken from human volunteers exposed to hydrogen peroxide mouthwashes^16^ established the general viability of the concept. Because serpin family members can comprise up to 2% of human plasma proteins^17^, we designed a clinical trial to test whether increases in S-glutathionylated serpins could be used as biomarkers to radiation exposure.

## Results

### Baseline characteristics

**Figure 1:**
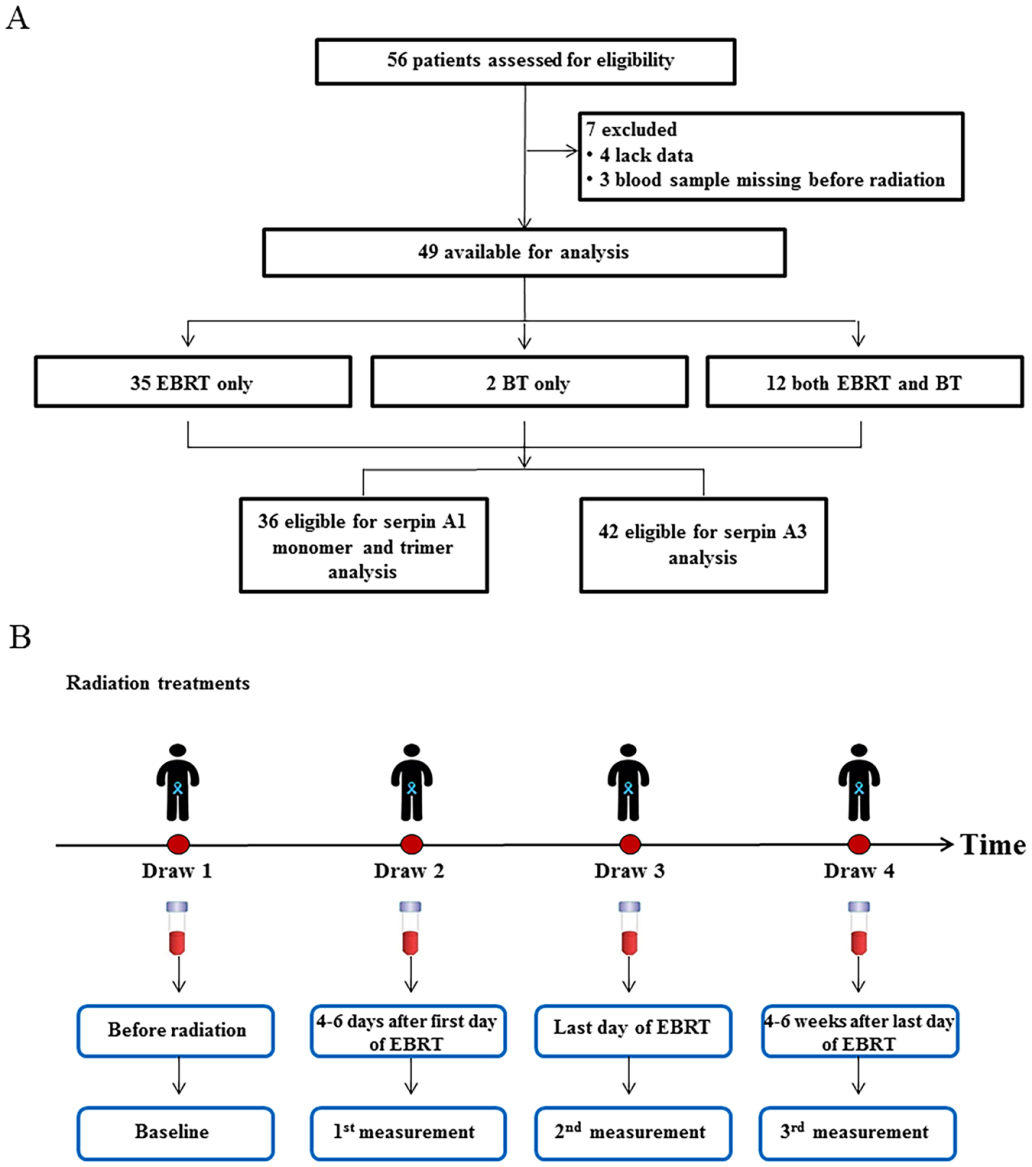
Analysis of S-glutathionylated serpin biomarkers in plasma from prostate cancer patients. (**A**) Patient flow diagram indicating patient selection criteria. (**B**) Study schema. At least 4 blood samples obtained from patients and the first draw was prior to either EBRT or BT and was consistently used as the comparative baseline; the second was 4–6 days after the first day of this treatment; the third was the last day of radiation and the fourth 4–6 weeks after the last day of treatment.

During the period from March 2013 to December 2016, 56 male prostate cancer patients were recruited to the trial (Fig. 1). By the end, seven were excluded for incomplete data sets, leaving 49 patients available for analysis. Of those, 35 patients received only EBRT (79.2 Gray (Gy) in 44 fractions over nine weels), 2 received only BT (low dose rate permanent seed implant, with prostate minimal peripheral dose target of 160 Gy), and 12 received both EBRT (45 Gy in 25 fractions over 5 weeks) and BT (low dose rate permanent seed implant with prostate minimal peripheral dose target of 100 Gy). 36 patients were eligible for the analysis of S-glutathionylated serpin A1 monomer and trimer; 42 for the analysis of S-glutathionylated serpin A3. Baseline characteristics of the patients in each treatment group are shown in Table 1, no differences were identified based on subject demographics. We used a variety of statistical analyses to identify radiation induced quantitative differences in S-glutathionylation of serpins A1 and A3.

**Table 1:** Baseline characteristics.

	EBRT	EBRT + BT	P-value
African American	19 (54.3%)	2 (16.7%)	
White	15 (42.9%)	9 (75.0%)	
Hormone Therapy Yes/No	22/13	10/2	
Age (years)	69.11 ± 1.23	67.17 ± 1.78	NS
Serpin A1 monomer	4.31 ± 0.56	4.06 ± 0.98	NS
Serpin A1 trimer	5.79 ± 0.81	6.76 ± 1.39	NS
Serpin A3	4.20 ± 0.54	4.47 ± 0.84	NS

### S-glutathionylated serpin A1

**Figure 2:**
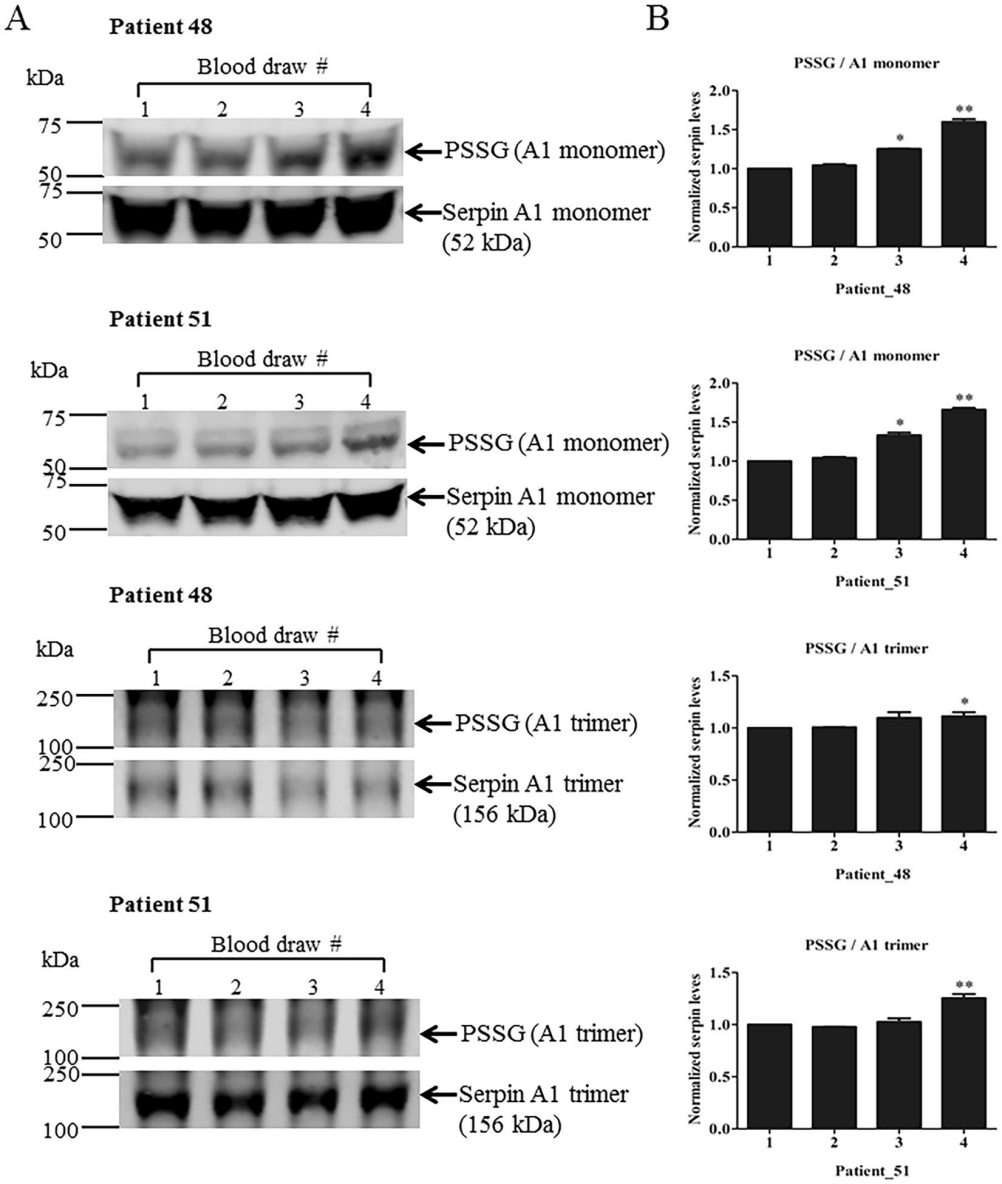
Effects of radiation therapy on formation of S-glutathionylated serpin A1 monomer and trimer in cancer patient plasma. (**A**) 20 μg of total pre- and post-treatment plasma proteins were separated by SDS-PAGE and evaluated by immunoblots. Unmodified and S-glutathionylated serpin A1 monomer and trimer protein levels were quantified with a two-channel (red and green) IR fluorescent Odyssey CLx imaging system (LI-COR). (**B**) Fold-changes in protein after radiation are expressed relative to pre-treated controls (with mean values set at 1). *p < 0.05, **p < 0.01 vs. the baseline control by one-way ANOVA and post-hoc tests. Uncropped images are presented in Supplementary Fig. S1. (x-axis, 1–4: blood draws 1–4).

Human plasma samples from 49 prostate cancer patients who underwent radiotherapy were analyzed for total and S-glutathionylated serpin A1 monomer and trimer (Fig. 2). Immunoblots showed that radiation caused no significant changes of levels of each of the unmodified serpin A1 species (monomer or trimer). The ratio of S-glutathionylated to unmodified serpin A1 monomer and trimer were relative to baseline values. Serpin S-glutathionylation profiles were patient-distinct, suggesting that each has a unique pattern of S-glutathionylation. For each serpin A1 monomer and trimer, we selected two representative immunoblots from two patients to show as examples (Fig. 2A). Patient 48 is EBRT + BT; patient 51 only EBRT. We did not find differences between the relative S-glutathionylated changes of serpin A1 monomer and/or trimer between EBRT and EBRT + BT treatments. The general trend was consistently increased levels of S-glutathionylated serpin A1 monomer and trimer compared with baseline after radiotherapy. Quantitation of S-glutathionylated serpin data from different blood draws is presented in Table 2.

**Table 2:** Serpin marker changes following radiation.

	EBRT				EBRT + BT			
	Draw 1	Draw 2	Draw 3	Draw 4	Draw 1	Draw 2	Draw 3	Draw 4
**Serpin A1 monomer**								
Median	5.90	5.98	5.41	5.84	5.27	6.62	6.15	6.24
25–75% Percentile	0.29–6.40	0.35–6.73	0.28–6.78	0.24–7.20	0.24–6.44	5.72–7.10	0.24–6.89	0.26–7.00
Changes		0.08	−0.49	−0.06		1.35	0.88	0.97
95% CI	3.16, 5.45	3.14, 5.68	2.81, 5.43	2.97, 6.61	1.81, 6.32	4.74, 8.22	1.45, 6.97	1.47, 7.09
**Serpin A1 trimer**								
Median	6.93	7.84	5.95	5.07	8.24	8.00	8.12	8.28
25–75% Percentile	1.00–9.11	1.02–9.02	0.81–9.10	0.78–7.76	0.78–9.59	6.96–9.04	0.88–10.56	1.01–10.6
Changes		0.91	−0.98	−1.86		−0.24	−0.12	0.04
95% CI	4.12, 7.45	4.20, 7.93	3.63, 7.61	3.25, 7.66	3.61, 9.90	−5.21, 21.21	3.00, 10.38	2.50, 10.51
**Serpin A3**								
Median	5.65	6.23	5.3	5.07	6.19	4.75	6.69	6.47
25–75% Percentile	0.30–6.62	0.30–6.94	0.28–6.42	0.34–6.49	0.28–6.44	1.32–6.49	0.49–7.35	0.31–7.27
Changes		0.58	−0.35	−0.58		−1.44	0.50	0.28
95% CI	3.10, 5.30	3.22, 5.77	2.66, 5.06	2.42, 5.08	2.58, 6.35	−0.27, 8.65	2.68, 7.48	2.18, 7.48

Changes are from baseline to post-radiation.

### Radiation induced temporal changes in S-glutathionylated serpin A1

**Figure 3:**
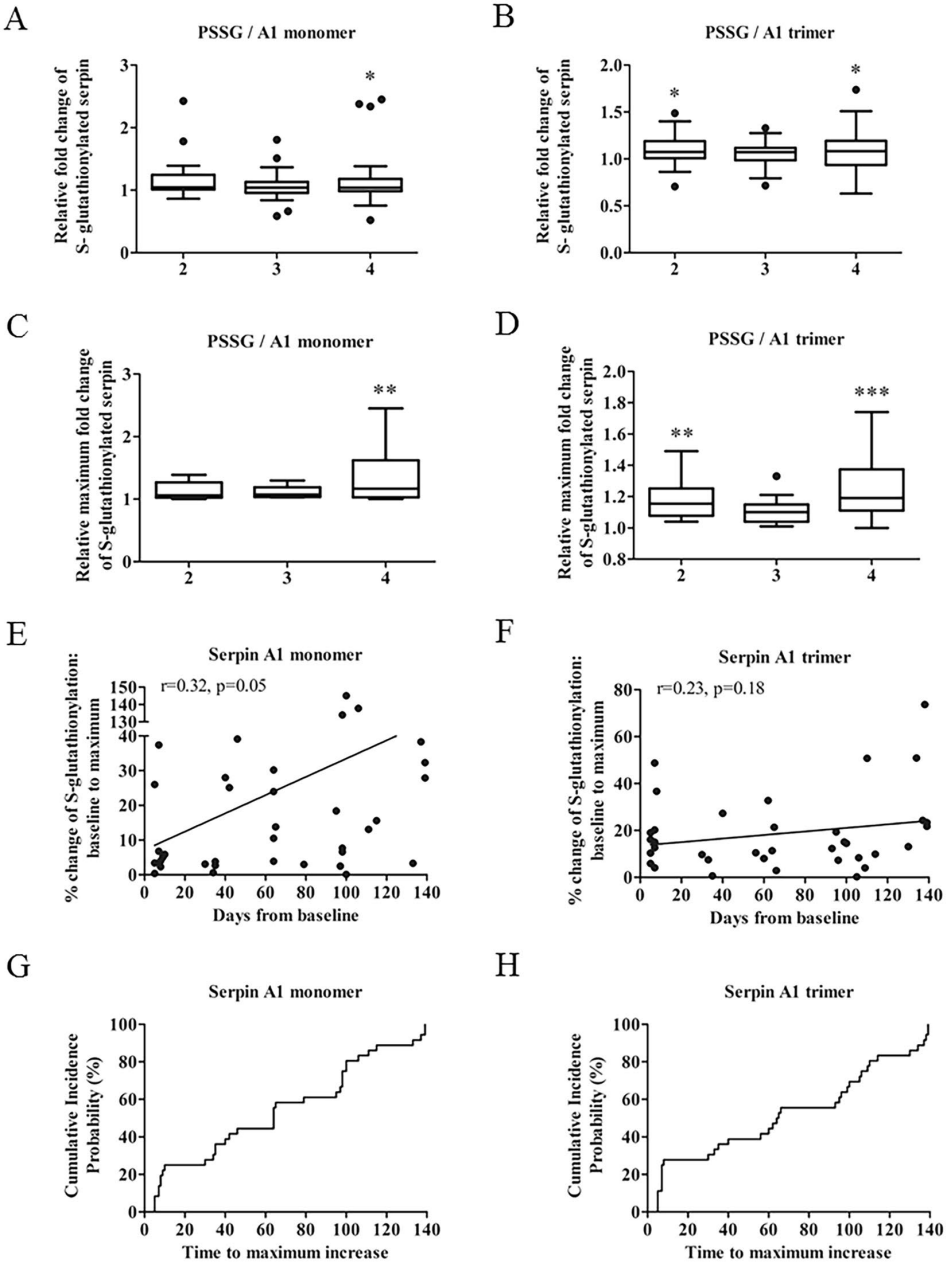
Enhanced levels of S-glutathionylation of serpin A1 in response to radiation. (**A**,**B**) Quantification of the fold changes in S-glutathionylated serpin A1 monomer and trimer after radiation. (**C**,**D**) Maximal fold changes of the S-glutathionylated serpin A1 monomer and trimer after radiation. Baseline values were set at 1. (**E,F**) Simple linear regression analyses between maximum percent increase of S-glutathionylated serpin A1 monomer and trimer and time after initiation of treatment (Serpin A1, n = 36, r = 0.32, p = 0.05; serpin A1 trimer, n = 36, r = 0.23, p = 0.18). (**G,H**) Cumulative incidence probability plots showing probability that maximum percent increases of S-glutathionylated serpin A1 monomer or trimer occurred before a given time point. *p < 0.05, **p < 0.01, ***p < 0.001 vs. the baseline control by one-way ANOVA and post-hoc tests. (x-axis, 2–4: blood draws 2–4).

There were no differences in the baseline characteristics between the EBRT and EBRT + BT groups before treatment (Table 1). For serpin A1 monomer, there was a statistically significant difference in the fold change of S-glutathionylated protein from three blood draws between groups, as determined by one-way ANOVA (F (3,121) = 2.708, p = 0.048). Post hoc comparisons using the Dunnett’s test indicated that the mean fold change of S-glutathionylated value for draw 4 (M = 1.17, SEM = 0.08) was significantly different from the baseline value (p < 0.05). For serpin A1 trimer, there was a statistically significant difference in the fold-change of S-glutathionylated protein from three blood draws between groups, as determined by one-way ANOVA (F (3,118) = 2.995, p = 0.033). Post hoc comparisons using the Dunnett’s test indicated that the mean fold change of S-glutathionylated values for draw 2 (M = 1.09, SEM = 0.03) and draw 4 (M = 1.09, SEM = 0.04) were significantly different from the baseline value (p < 0.05) (Table 3 (left) and Fig. 3A,B).

**Table 3:** Comparative changes in S-glutathionylated serpins following radiation.

	Median	25–75% Percentile	Fold change	95% CI	P-value	Median	25–75% Percentile	Maximum fold change	95% CI	P-value
**Serpin A1 monomer**					P = 0.04					P = 0.005
Draw 2	1.05	1.01–1.25	0.16	1.04, 1.28	NS	1.06	1.03–1.27	0.14	1.05, 1.23	NS
Draw 3	1.04	0.96–1.13	0.06	0.98, 1.14	NS	1.07	1.04–1.19	0.11	1.03, 1.19	NS
Draw 4	1.04	0.98–1.18	0.17	1.00, 1.34	*	1.17	1.03–1.62	0.41	1.10, 1.73	**
**Serpin A1 trimer**					P = 0.03					P = 0.0002
Draw 2	1.08	1.01–1.19	0.1	1.03, 1.17	*	1.16	1.08–1.25	0.19	1.10, 1.27	**
Draw 3	1.07	0.99–1.12	0.06	1.01, 1.10	NS	1.1	1.04–1.15	0.11	1.05, 1.17	NS
Draw 4	1.08	0.94–1.19	0.09	1.01, 1.18	*	1.19	1.11–1.38	0.25	1.12, 1.38	***
**Serpin A3**					P = 0.08					P = 0.02
Draw 2	1.06	1.00–1.21	0.12	1.05, 1.20	NS	1.14	1.05–1.31	0.22	1.11, 1.33	NS
Draw 3	1.09	0.98–1.14	0.14	1.00, 1.27	NS	1.12	1.06–1.30	0.33	0.97, 1.68	*
Draw 4	1.05	0.96–1.21	0.12	1.01, 1.23	NS	1.19	1.12–1.46	0.26	1.14, 1.38	NS

Values were statistically significant if p < 0.05 (*), P < 0.01 (**), p < 0.001 (***).

### Maximal changes in S-glutathionylated serpin A1

Using a one-way ANOVA analysis, there was a statistically significant difference in the extent of the maximum fold change in S-glutathionylated serpin A1 monomer of three variables between groups (F (3, 46) = 4.724, p = 0.005). Post hoc comparisons using the Dunnett’s test indicated that the mean fold change of S-glutathionylated values for draw 4 (M = 1.33, SEM = 0.12) was significantly different from baseline (p < 0.01).

For the serpin A1 trimer, there was a statistically significant difference in the maximum fold change of S-glutathionylated values for three blood draws between groups, as determined by one-way ANOVA (F (3, 45) = 8.066, p = 0.0002). Post hoc comparisons using the Dunnett’s test indicated that the mean fold change of S-glutathionylated values for draw 2 (M = 1.19, SEM = 0.03) and draw 4 (M = 1.26, SEM = 0.06) were significantly different from the baseline value (p < 0.01; p < 0.001) (Table 3 (right), Fig. 3C,D).

### Time to maximal increase in S-glutathionylated serpin A1

Duration of radiation treatments was found to correlate positively with maximum percent increase of S-glutathionylated serpin A1 monomer (r = 0.32, p = 0.05) and trimer (r = 0.23, p = 0.18) (Fig. 3E,F). When modeled with simple linear regression, we found that S-glutathionylated serpin A1 monomer and trimer significantly correlated with draw 4 (monomer: r = 0.44, p = 0.01; trimer: r = 0.60, p = 0.0009) (Table 4).

**Table 4:** Analysis of comparative S-glutathionylated serpin increases with time.

Dependent variables	Independent variables	Draw 2	Draw 3	Draw 4
PSSG Serpin A1 monomer	Days from baseline (Draw 1)	n = 28, r = 0.18	n = 31, r = 0.35	n = 30, r = 0.44*
PSSG Serpin A1 trimer		n = 27, r = 0.22	n = 31, r = 0.24	n = 28, r = 0.60***
PSSG Serpin A3		n = 33, r = −0.47	n = 36, r = 0.13	n = 33, r = 0.34

Sample sizes (n) for each treatment and Pearson correlation coefficient (r) are given for each regression, as dependent and independent variables. Values were statistically significant if p < 0.05 (*), P < 0.01 (**), p < 0.001 (***).

### Time to event with the maximum percent increase of serpin A1 monomer and trimer S-glutathionylation

Using time-to-event data analyses, we calculated cumulative incidence functions for both S-glutathionylated serpin A1 monomer and trimer (Fig. 3G,H). On or before day 64, 50% of the patients displayed maximum percent increases in the ratios of S-glutathionylated serpin A1 monomer and trimer.

### S-glutathionylated serpin A3

**Figure 4:**
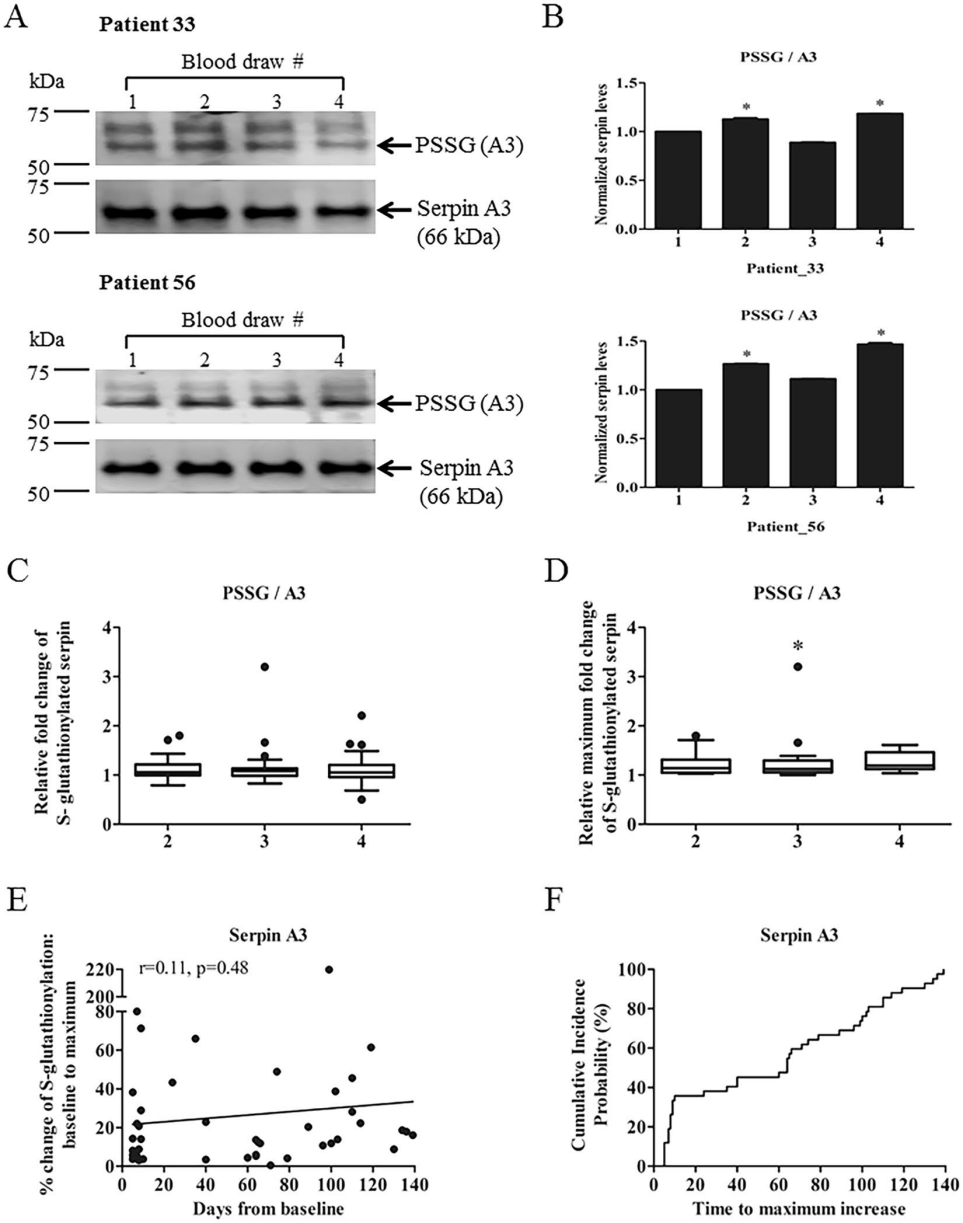
Enhanced levels of S-glutathionylated serpin A3 in response to radiation. (**A**) 20 μg of pre- and post-treatment plasma proteins were separated by SDS-PAGE and evaluated by immunoblots. Unmodified and S-glutathionylated serpin A3 levels were detected with a two-channel (red and green) IR fluorescent Odyssey CLx imaging system (LI-COR). (**B**) Fold changes after radiation therapy relative to pre-treated controls (with mean values set at 1) were quantified by Image J software. (**C**) Fold changes of S-glutathionylated serpin A3 after radiation treatments. (**D**) Maximal fold changes of S-glutathionylated serpin A3 after radiation treatments. All data were expressed relative to baseline controls with mean values set at 1. (**E**) Simple linear regression between maximum percent increases of S-glutathionylated serpin A3 and corresponding time points (days from baseline) (n = 42, r = 0.11, p = 0.48). (**F**) Cumulative incidence probability plots show the probability that maximum percent increases of S-glutathionylated serpin A3 occurred before a given time point. *p < 0.05 vs. the baseline control by one-way ANOVA and post-hoc tests. Uncropped images are presented in Supplementary Fig. S1. (x-axis, 1–4: blood draws 1–4).

Immunoblots showed that unmodified serpin A3 levels were similar in all plasma samples measured. Ratios of S-glutathionylated to unmodified serpin A3 were used to compare the S-glutathionylation patterns and two patient samples are shown (Fig. 4A,B). Patients 33 and 56 were EBRT only. We found no significant differences between the relative changes of S-glutathionylated serpin A3 from EBRT and EBRT + BT groups. The general trend was increased levels of S-glutathionylated serpin A3 after radiotherapy. The overall S-glutathionylated serpin A3 values from different blood draws are presented in Table 2. There was a statistically significant difference for the maximum fold change of S-glutathionylated values from three blood draws between groups, as determined by one-way ANOVA (F (3, 56) = 3.293, p = 0.027). Post hoc comparisons using the Dunnett’s test indicated that the mean fold change of the maximum S-glutathionylated value for draw 2 (M = 1.33, SEM = 0.16) was significantly different from the baseline value (p < 0.05). (Table 3, Fig. 4C,D).

The duration of the radiation treatments positively correlated with maximum percent increase of S-glutathionylated serpin A3 (r = 0.11, p = 0.48) (Fig. 4E). On or before 64 days, 50% of patients displayed their maximum percent increase of S-glutathionylated serpin A3 by time-to-event data analysis (Fig. 4F).

## Discussion

At this time there are no other standard serum markers for radiation exposure. Disease response markers, such as PSA would not be reliable as short-term markers in this study since it is a biomarker of prostate cancer, not of radiation exposure or dose. While is usually decreases after radiation, PSA is released as the prostate cancer cells die, which can take months or years to occur after radiation exposure and varies a great deal among patients. In one study of 841 men treated with external beam radiotherapy, PSA half-life after radiation ranged from very short (0.5 months, rapidly declining) to long (>9 months, slowly declining). And PSA half-life did not correlate with disease outcome^18^.

In this clinical trial, we sought to determine the feasibility of measuring changes in circulating levels of S-glutathionylated serpins as plausible biomarkers for exposure to ROS generated by radiation^3,19^. Because many classes of anticancer drugs result in either the direct or indirect creation of free radicals and/or ROS, our tenet is that radiation can be a potential predictor for a number of different types of cancer modalities and may be used in the future as a predictive biomarker in clinical trials. Moreover, there is a current need for human biomarkers for radiation/nuclear medical countermeasures. Our previous preclinical data showed that in mice treated with anticancer drugs, there was a dose and time response correlation with blood levels of S-glutathionylated serpins^15^. This principle was extended into a trial using buccal cell sampling after exposure to hydrogen peroxide containing mouthwashes^16^. The present clinical trial shows that quantitative increases in S-glutathionylated serpins A1 and A3 can be used as blood biomarkers reflecting time of exposure to radiation. Because the post-translational modification involves thiol groups on susceptible cysteine residues, these modified proteins primarily represent surrogate measures of the whole-body exposure to the ROS caused by radiation effects on biological systems. Across all patients tested, there was no significant difference in the plasma levels of unmodified serpin A1 (monomer or trimer) or A3. It was only following exposure to radiation that the statistically significant increases in S-glutathionylated serpins were found. Indeed, prior to radiation, essentially none of the post-translationally modified forms were found in blood.

Polymeric forms of serpins have been associated with various degrees of metastability that can impact their biological activities and can even contribute to serpinopathies^20^. Crystal structures of wild type trimeric serpins have properties that suggest they are capable of forming oligomers of twisted linear or near globular structures^21^. However, there are presently no indications if cysteine residues in the serpin molecules contribute to these folding patterns. As such, our present clinical results do not allow for prediction of the precise importance of the balance of monomer and trimer and/or their S-glutathionylated derivatives.

In conclusion, the results of the present trial provide evidence supporting the utility of plasma S-glutathionylated serpins as viable pharmacodynamic biomarkers for cumulative exposure to ionizing radiation. This post-translational modification could prove useful in other clinical settings, such as radiation/nuclear medical countermeasures or where ROS causing therapeutics/toxins, alter redox-homeostasis. We recognize that future efforts may establish an increased sensitivity of detection of these post-translationally modified proteins by establishing methods for MS/MS analysis of peptide fragments of the S-glutathionylated serpins.

## Methods

### Study design and study participants

Patients who were on the Radiation Oncology outpatient service with a diagnosis of prostate cancer were contacted regarding study participation after clearance from the patient’s attending physician and a co-investigator on the trial. 56 prostate cancer patients ages between 18 and 75 were enrolled in the trial. Blood samples were obtained through an approved IRB protocol (HR # 19295), and samples were segregated on the basis of age (18–40; or 41+ years) and race (while no races were excluded, demographics resulted in primarily Caucasian or African American). Blood was drawn from 56 patients, however, 7 were excluded from the final analysis based upon non-compliance based upon timing of the samples taken leaving samples from 49 patients available for data analysis.

### Blood draws from patients

If the patient was not subject to a blood draw for standard laboratory studies, an extra venopuncture (8–10 mL) was performed and placed in a vacutainer with ethylenediaminetetraacetic acid (EDTA). The tube was gently tilted (x8) to mix blood and EDTA. Samples were then catalogued and placed on insulated ice (small cooler with tissue paper separating direct contact between ice and tubes). Initial pre-treatment blood samples were drawn during a routine visit, prior to radiotherapy start date, independent of the type of radiation treatment. Additional subsequent blood draws were collected throughout treatment, as described below.

For patients who received EBRT only, or EBRT and BT together, there were at least 4 blood samples obtained from patients (Fig. 1). (Some patients who received hormonal therapy could have one more draw after 2–3 months of hormonal therapy, before radiation treatment). The first draw was prior to either EBRT or BT and was consistently used as the comparative baseline; the second was 4–6 days after the first day of treatment; the third was the last day of radiation and the fourth 4–6 weeks after the last day of treatment. For patients who received BT only, there was 1 draw before BT and a second draw 4–6 weeks after treatment. For patients receiving hormonal therapy an additional blood draw was taken prior to radiotherapy (EBRT or BT) but after 2–3 months of hormonal therapy in order to distinguish the effects of hormonal and radiotherapy.

During EBRT, blood was drawn at the conclusion of five radiotherapy treatments, within 30 minutes but no later than 2 hours following radiotherapy. A further blood draw occurred on the final day of treatment, again 30 minutes to 2 hours following radiotherapy. Patients that received BT prior to EBRT had a blood draw 4 weeks after the BT procedure at their post-implant follow-up appointment, but not at the conclusion of the five treatments of EBRT. This group is also subject to a blood draw after the final day of EBRT.

### Processing samples for storage

Samples were processed within two hours of harvesting. They were centrifuged at 3000 rpm for 10 minutes at 4 °C in a cold room and aliquot 1.5 mL of plasma was aliquoted into 3 labelled 1.8 mL cryovials. These were stored at −80 °C to arrest protease activity.

### Immunoblotting

Plasma protein concentrations were assessed using bicinchoninic acid (Pierce, Rockford, IL). 20 μg of total pre- and post-treatment plasma proteins were resolved in an SDS-loading buffer (80 mM tris-HCl, pH 6.8, 2% SDS, 10% glycerol, 0.02% bromophenol blue, 5 mM tris (2-carboxyethyl) phosphine) and heated at 95 °C for 5 min. Equal amounts of protein were electrophoretically separated by SDS-PAGE (BioRad) and transferred onto low fluorescent polyvinylidene fluoride membranes (Millipore, Billerica, MA) or nitrocellulose membranes (BioRad) by the Trans-Blot Turbo Transfer System (BioRad). PVDF or nitrocellulose membranes were incubated in the Odyssey blocking buffer (LI-COR, Lincoln, NE) for 1 h to reduce non-specific binding and then probed with goat polyclonal anti-serpin A1, goat polyclonal anti-serpin A3 or mouse monoclonal anti-GSH primary antibodies (diluted in Odyssey blocking buffer) at 4 °C overnight. Unmodified serpin A1 monomers and trimers, A3, or their S-glutathionylated couterparts) were detected using infrared (IR) fluorescence IRDye secondary antibodies (LI-COR) at a dilution of 1:15,000 and imaged with a two-channel (red and green) IR fluorescent Odyssey CLx imaging system (LI-COR) and quantified with Image Studio 4.0 software (LI-COR).

### Fold and percent change of S-glutathionylated serpins

Three biomarker proteins were measured from each blood sample; serpin A1 in both its monomer and trimer forms, serpin A3. Ratios of unmodified and S-glutathionylated of each were used as dependent variables in the analysis.

For each patient and each of 3 serpin values, we quantified each S-glutathionylated serpin value from all blood draws and calculated fold changes and percent changes using pretreatment samples as the denominators. We also calculated fold change and percent change values for the maximal S-glutathionylated serpin values and the first draw.

$${\rm{Fold}}\,{\rm{change}}:\frac{{\rm{subsequent}}\,{\rm{blood}}\,\mathrm{draws}\,}{{\rm{Baseline}}};{\rm{Percent}}\,{\rm{change}}:\frac{({\rm{subsequent}}\,{\rm{blood}}\,{\rm{draws}}-\mathrm{Baseline}\,)\,}{{\rm{Baseline}}}\times 100.$$

### Data analysis and statistics

Using the D’Agostino and Pearson omnibus and the Shapiro-Wilk normality tests, most variables were normally distributed. The median and 25–75% percentiles were determined for EBRT and BT (Table 1). Between groups comparisons were performed by one-way analysis of variance (ANOVA) and post-hoc tests Dunnett and Tukey tests. Simple linear regressions and Pearson correlations were used to determine the significance of correlations between time and the maximum percent changes of S-glutathionylated serpins. All analyses were performed with R statistical software version 3.5.0 and GraphPad Prism 6.0. A p-value of <0.05 was considered significant and values were expressed as the means ± *SEM*.

### Ethics statement

All study participants provided signed informed consent, and this study was approved by the Institutional Review Board for Human Research (IRB) and the Office of Research Integrity (ORI) of Medical University of South Carolina, Charleston, South Carolina, USA. The methods were carried out in accordance with the approved guidelines.

## Supplementary information

Supplementary Figure S1

## Data Availability

The datasets generated during the current study are available from the corresponding author on reasonable request.
