# Supplementary material for: S-Glutathionylated Serine Proteinase Inhibitors as Biomarkers for Radiation Exposure in Prostate Cancer Patients
Source: Sci Rep. 2019 Sep 24;9:13792. doi: 10.1038/s41598-019-50288-9 (PMC6760651; doi:10.1038/s41598-019-50288-9)

**S-Glutathionylated Serine Proteinase Inhibitors as Biomarkers for Radiation Exposure in Prostate Cancer Patients.**

Leilei Zhang^1^, Jie Zhang^1^, Zhiwei Ye^1^, Yefim Manevich^1^, Danyelle M. Townsend^2^, David T. Marshall^3^ and Kenneth D. Tew^1*^.

Departments of ^1^Cell and Molecular Pharmacology and Experimental Therapeutics, ^2^Pharmaceutical and Biomedical Sciences, and ^3^Radiation Oncology, Medical University of South Carolina, Charleston, South Carolina.

**^*^Corresponding author:** Kenneth D. Tew, Department of Cell and Molecular Pharmacology and Experimental Therapeutics, Medical University of South Carolina, 171 Ashley Avenue, Charleston, South Carolina 29425, USA. Tel.: +1 843-792-2514. Fax: 843-792-2475. Email: [tewk@musc.edu](mailto:tewk@musc.edu)

**Supplementary Figure S1.** Uncropped images for which boxed areas are shown in the main manuscript. (A) Corresponding to Figure 2A. (B) Corresponding to Figure 2A. (C) Corresponding to Figure 4A.


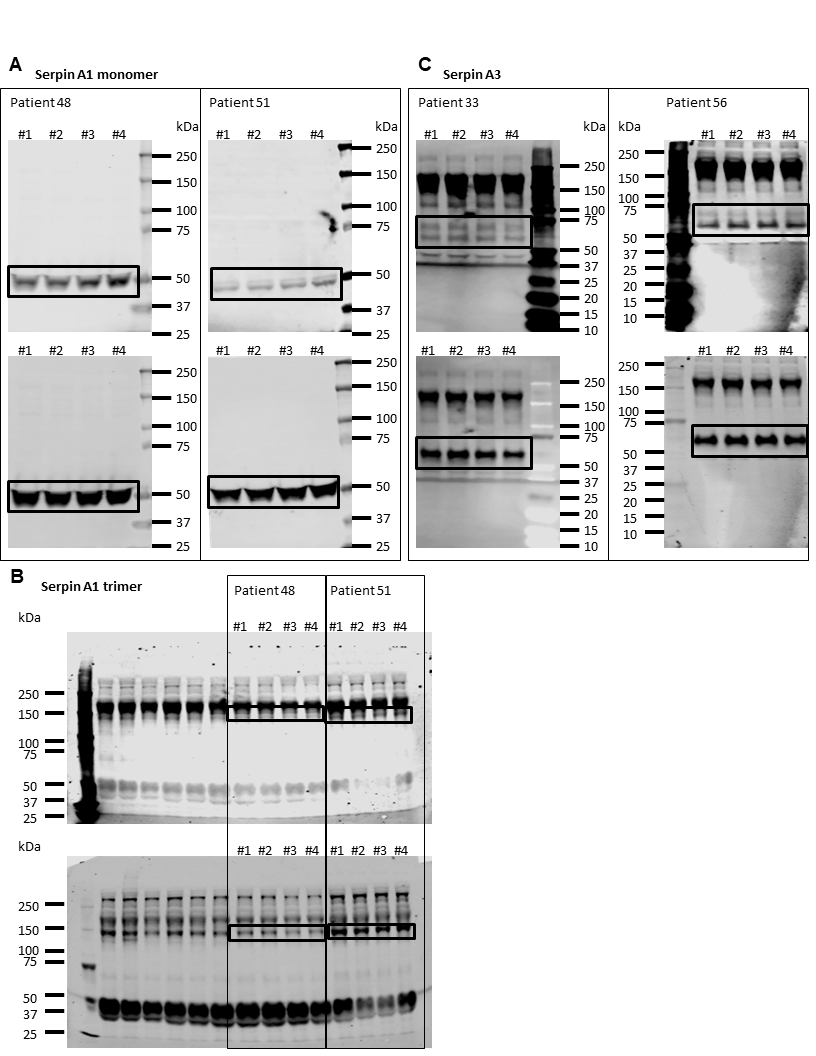

Supplement: Supplementary file 1 — Supplementary Figure S1 [file 41598_2019_50288_MOESM1_ESM.docx]
